# High-accuracy lung sound classification for healthy versus unhealthy diagnosis using artificial neural network

**DOI:** 10.3389/fbioe.2025.1583416

**Published:** 2025-07-02

**Authors:** Weiwei Zhang, Xinyu Li, Qiao Liu, Xiangyang Zheng, Yisu Ge, Xiaotian Pan, Yu Zhou

**Affiliations:** ^1^ Infectious Disease Department, The Third Affiliated Hospital of Wenzhou Medical University, Wenzhou, China; ^2^ China Telecom Corporation Limited Zhejiang Branch, Hangzhou, China; ^3^ Information Technology Center, Wenzhou Medical University, Wenzhou, China; ^4^ School of Data Science and Artificial Intelligence, Wenzhou University of Technology, Wenzhou, China; ^5^ College of Computer Science and Artificial Intelligence, Wenzhou University, Wenzhou, China; ^6^ Institute of Intelligent Media Computing, Hangzhou Dianzi University, Hangzhou, China; ^7^ Shangyu Institute of Science and Engineering Co. Ltd., Hangzhou Dianzi University, Shaoxing, China

**Keywords:** machine learning, pulmonary disease classification, lung sounds, electronic stethoscope, multi-layer perceptron, feature extraction

## Abstract

**Introduction:**

In recent years, advancements in machine learning and electronic stethoscope technology have enabled high-precision recording and analysis of lung sounds, significantly enhancing pulmonary disease diagnosis.

**Methods:**

This study presents a comprehensive approach to classify lung sounds into healthy and unhealthy categories using a dataset collected from 112 subjects, comprising 35 healthy individuals and 77 patients with various pulmonary conditions, such as asthma, heart failure, pneumonia, bronchitis, pleural effusion, lung fibrosis, and chronic obstructive pulmonary disease (COPD), grouped as unhealthy. The dataset was obtained using a 3M Littmann® Electronic Stethoscope Model 3,200, employing three types of filters (Bell, Diaphragm, and Extended) to capture sounds across different frequency ranges. We extracted five key audio features—Spectral Centroid, Power, Energy, Zero Crossing Rate, and Mel-Frequency Cepstral Coefficients (MFCCs)—from each recording to form a feature matrix. A Multi-Layer Perceptron (MLP) neural network was trained for binary classification.

**Results:**

The MLP neural network achieved accuracies of 98%, 100%, and 94% on the training, validation, and testing sets, respectively. This partitioning ensured the model’s robustness and accuracy.

**Discussion:**

The high classification accuracy achieved by the MLP neural network suggests that this approach is a valuable decision-support tool for identifying healthy versus unhealthy lung sounds in clinical settings, facilitating early intervention while maintaining computational efficiency for offline implementation. The combination of detailed feature extraction and an optimized MLP neural network resulted in a reliable method for automated binary classification of lung sounds.

## 1 Introduction

The accurate diagnosis of pulmonary diseases is crucial for effective treatment and management, yet it remains a significant challenge in clinical practice. Traditional methods rely heavily on the expertise of healthcare professionals to interpret auscultation sounds, which can be subjective and prone to human error. The stethoscope is an essential diagnostic instrument used in clinical studies to diagnose pulmonary disorders (PDs) and cardiac valve disorders (Nishio et al., 2021). Since the advent of the digital stethoscope, patients’ lung sound (LS) data may be continuously recorded, allowing for the automated diagnosis of several PDs (Khan and Pachori, 2022). Conditions such as COPD, heart attacks, asthma, pneumonia, bronchitis, and lung fibrosis may be detected by listening to the signals produced by the lungs (Rao et al., 2018; Lehrer, 2018). It is possible to diagnose certain lung disorders by listening for abnormal sounds such wheezes, crackles, or rhonchi (Zulfiqar et al., 2021). According to statistics on mortality and worldwide deaths, lung diseases are now the third leading cause of death worldwide. Chronic obstructive pulmonary disease (COPD) ranks as the world’s fourth most deadly disease, and the number two killer in India. Among all causes of death worldwide, it is predicted to overtake tobacco use by 2030 (Institute for Health Metrics and, 2021). It ranks as the fourth most prevalent reason for women to be hospitalized and affects between five and nineteen percent of those over above the age of forty. Using a stethoscope to listen to the patient’s breathing has long been a standard method for diagnosing respiratory issues by both specialists and family doctors. Some pulmonary disorders may be better understood by listening for lung sounds like crackles or wheezes (Sarkar et al., 2015; Rocha et al., 2018). Support vector machine (SVM) is used by the writers in (Bokov et al., 2016) to identify wheezes. In order to identify features in lung sounds, they use the short-time Fourier transform (STFT). The study documented in (Kandaswamy et al., 2004) reveals the distribution of wavelet coefficients, utilizing an artificial neural network (ANN) to detect signals from lung sounds. Wavelet analysis has been applied to extract data from the audio signals mentioned in references (Liu and Xu, 2014; G¨og¨u et al., 2015). Additionally, the researchers in reference (Shi Y. et al., 2019) have minimized the dimensions of the wavelet coefficients through linear discriminant analysis. Meanwhile, reference (Liu et al., 2006) employs wavelet packet decomposition to analyze the lung sound energy across various frequency bands. The research in (Jin et al., 2014) focuses on examining the temporal characteristics of repetitive narrow-band signals to differentiate between normal and abnormal respiratory sounds. Exploration of wavelet decomposition alongside linear predictive cepstral coefficients (LPCC) is covered in (Azmy, 2015). The effectiveness of Support Vector Machine (SVM) and k-nearest neighbor (KNN) classifiers in diagnosing respiratory ailments is discussed in (Palaniappan et al., 2014). A Gaussian mixture model has been developed in (Haider et al., 2014) to separate typical from atypical lung sounds. The primary investigation of (Yao et al., 2016) centers on utilizing a genetic back-propagation neural network for lung sound analysis.

On a different note, these signals can be detected through a multi-channel linear parametric technique for analyzing lung sounds as elaborated in (Santiago-Fuentes et al., 2017). To achieve improved recognition accuracy, a neural network is utilized during the classification process. To distinguish between individuals with asthma and those without using 4-channel data, the researchers cited in (Islam et al., 2018) extract specific statistical features from lung sounds which are then input into ANN and SVM classifiers. Recent developments in deep learning have paved new paths for solving this issue. A strategy involving a convolutional neural network (CNN) to segregate various lung sound types is elaborated in (Aykanat et al., 2017). This approach utilizes a two-layer CNN trained on mel frequency cepstral coefficients (MFCC). The findings demonstrate that the CNN’s detection capabilities surpass those of the Support Vector Machine (SVM) technique. Employing Fourier transform analysis, the team in (Chen et al., 2017) evaluates the transient time-frequency features of lung sounds. They use a CNN with dual-layer full connections to sort the lung sounds into three distinct groups. Furthermore, the researchers in (Chen et al., 2019) have investigated how deep residual networks, when paired with an accurate S-transform, can discern normal, crackling, and wheezing sounds. Tripathy et al. (Tripathy et al., 2022) apply a statistical wavelet transform with set boundary points to examine the lung sounds. Classifiers such as SVM, Random Forest, Extreme Gradient Boosting, and Light Gradient Boosting Machine (LGBM) are typically used for synthetically diagnosing Parkinson’s disease. By utilizing ensemble classifiers and the empirical mode decomposition (EMD) technique, it is possible to differentiate between chronic and non-chronic conditions (Khan and Pachori, 2022). Additionally, Fraiwan et al. (Fraiwan L. et al., 2021) investigate homogeneous ensemble learning techniques for the multi-class classification of respiratory conditions. They utilize features based on spectrograms including Shannon entropy, logarithmic energy entropy, and spectral entropy to represent lung sound signals. Moreover, deep learning methods are explored for lung sound data classification in (Basu and Rana, 2020). Features such as spectrograms, MFCCs, and chromatograms are analyzed by the researchers in (Tariq et al., 2019) for classification using a 2D-CNN. Aiming to establish an automated system capable of diagnosing Parkinson’s disease (PD), a deep learning-based VGGish model is proposed in (Shi L. et al., 2019), although only three PD cases were included in their study.

In this study, we present an approach to the automated classification of lung sounds for distinguishing between healthy and unhealthy pulmonary conditions. The model performs binary classification, categorizing lung sounds as healthy or unhealthy, with the unhealthy category encompassing various pulmonary conditions, rather than identifying specific diseases. Using a dataset collected from 112 subjects, we extracted key audio features from recordings obtained through an electronic stethoscope. These features were then used to train a Multi-Layer Perceptron (MLP) neural network to classify lung sounds into two categories: healthy and unhealthy. The dataset was divided into training, validation, and testing sets, and the neural network achieved high classification accuracy across these subsets. The remainder of this paper is structured as follows: Section 2 details the dataset and preprocessing steps. Section 3 describes the feature extraction methods employed. Section 4 outlines the architecture and training process of the MLP neural network. Section 5 presents the results of our classification experiments, including accuracy metrics and confusion matrices. Finally, Section 6 discusses the implications of our findings and potential future directions for research.

## 2 Dataset and preprocessing

The dataset used in this study, which includes lung sound recordings from both healthy individuals and patients with various pulmonary conditions, was sourced from a publicly available repository detailed in reference (Fraiwan M. et al., 2021). This repository provides comprehensive data essential for the analysis and classification of lung sounds. The dataset used in this study comprises lung sound recordings from 112 subjects, including both healthy individuals and patients with various pulmonary conditions. These recordings were acquired using a 3M Littmann^®^ Electronic Stethoscope Model 3,200, which provides high-fidelity audio data crucial for accurate analysis. The stethoscope was placed on multiple chest locations to capture sounds from different lung regions, using three types of filters: Bell, Diaphragm, and Extended. Each filter emphasizes specific frequency ranges, ensuring comprehensive coverage of lung sounds.

The study involved 112 subjects, with a mean age of 50.5 years (±19.4), ranging from 21 to 90 years. The cohort included 43 females and 69 males. The subjects were categorized into healthy and unhealthy groups, with 35 healthy individuals and 77 patients diagnosed with various pulmonary diseases such as asthma, heart failure, pneumonia, bronchitis, pleural effusion, lung fibrosis, and chronic obstructive pulmonary disease (COPD)​.

**FIGURE 1:**
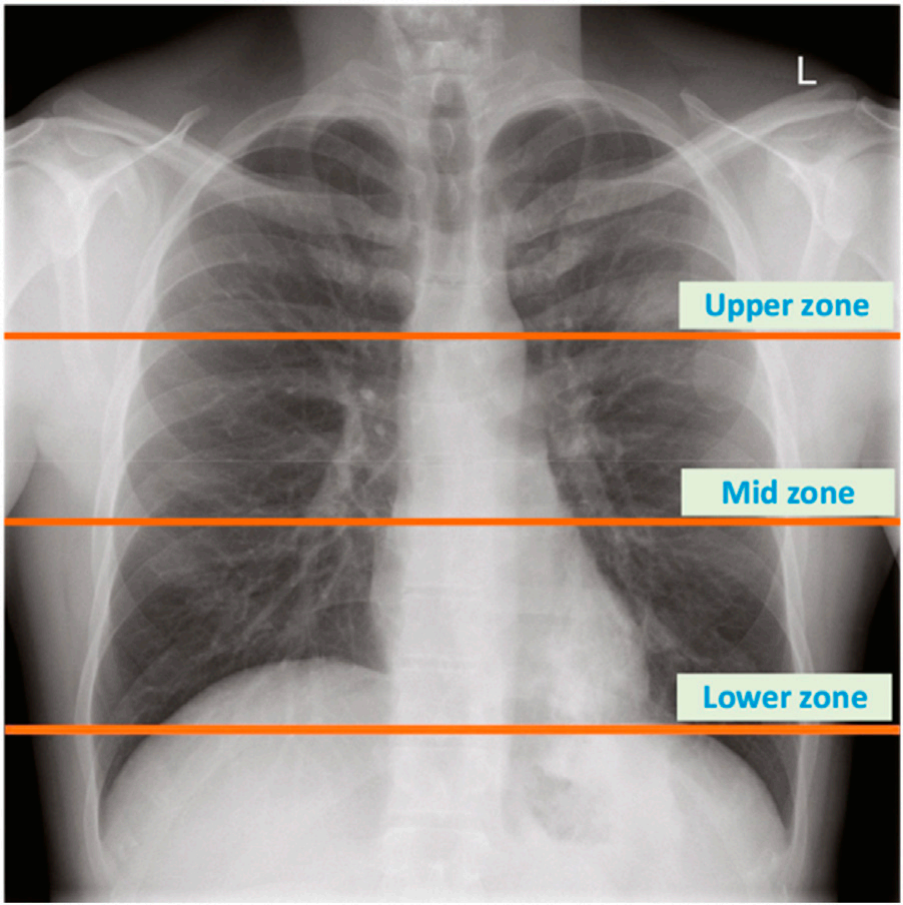
The location of chest zones used to record lung sounds (Fraiwan M. et al., 2021).

The dataset encapsulates a diverse range of pulmonary conditions. Specifically, it includes 35 healthy subjects, 32 subjects with asthma, five subjects with pneumonia, nine subjects with chronic obstructive pulmonary disease (COPD), three subjects with bronchitis, 21 subjects with heart failure, five subjects with lung fibrosis, and two subjects with pleural effusion. These conditions were diagnosed by healthcare professionals and recorded using the electronic stethoscope at various chest locations, as depicted in Figure 1. The recordings varied in duration from 5 to 30 s, ensuring at least one complete respiratory cycle was captured in each recording. The Bell filter emphasizes sounds in the range of 20–200 Hz, making it suitable for heart sounds. The Diaphragm filter covers a broader range of 100–500 Hz, while the Extended filter spans 50–500 Hz, capturing comprehensive lung sound frequencies.

Preprocessing of the audio data involved several key steps to ensure the quality and consistency required for subsequent analysis. The primary preprocessing steps included filtering, normalization, and segmentation of the audio signals.

Filtering: Each recording was subjected to three types of filters—Bell, Diaphragm, and Extended. These filters are essential for emphasizing different frequency ranges in the lung sounds:

Bell Filter: This filter amplifies sounds in the 20–1,000 Hz range, with a particular emphasis on low-frequency sounds between 20–200 Hz. It is particularly effective for capturing heart sounds.

Diaphragm Filter: This filter covers a wider frequency range of 20–2,000 Hz, emphasizing sounds between 100–500 Hz, making it suitable for lung sound analysis.

Extended Filter: This filter spans the 20–2,000 Hz range but emphasizes frequencies between 50–500 Hz, providing a balanced capture of both heart and lung sounds.

Normalization: To ensure consistency in the amplitude of the signals, each audio recording was normalized. This step is crucial for eliminating variations in signal strength due to differences in recording conditions or subject characteristics.

**FIGURE 2:**
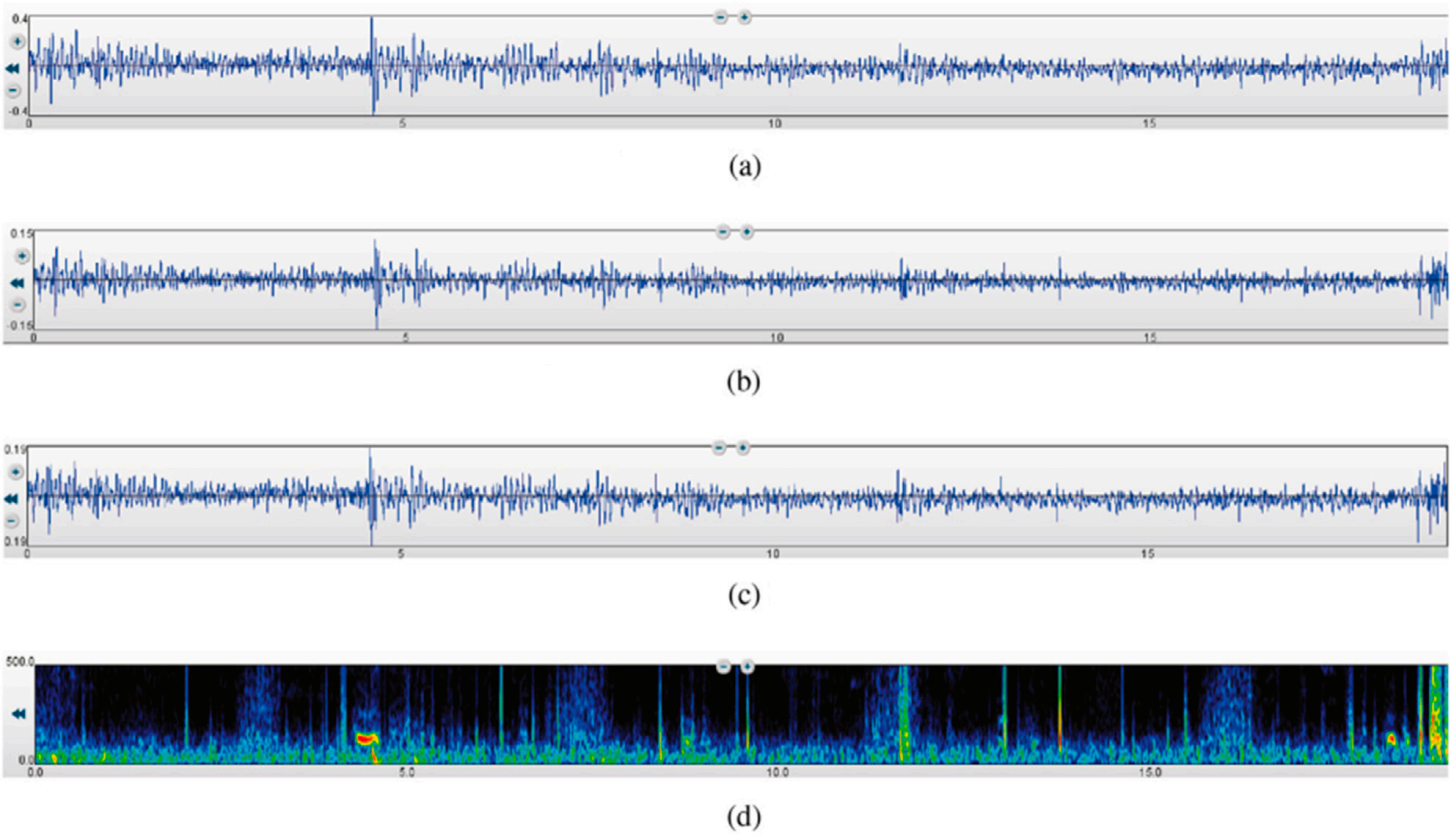
A 19-s recording of respiratory lung sound using the three filters and the spectrogram (Fraiwan M. et al., 2021). **(a)** Bell mode filtration. **(b)** Diaphragm mode filtration. **(c)** Extended mode filtration. **(d)** Spectrogram.

Segmentation: The recordings were segmented into consistent time frames to standardize the input for further analysis. This segmentation ensures that each segment contains a complete respiratory cycle, facilitating accurate feature extraction and analysis. The rigorous preprocessing steps ensured that the data fed into the subsequent analysis was of high quality, enabling effective classification of lung sounds into healthy and unhealthy categories. These steps are essential for leveraging machine learning techniques to their full potential in medical diagnostics. Figure 2 illustrates a 19-s recording of respiratory lung sound processed using three different filters—Bell, Diaphragm, and Extended—and the corresponding spectrogram, showcasing the variations in lung sound frequencies captured by each filter.

To ensure robust lung sound classification in the presence of variability from patient positioning, microphone placement, and environmental noise, our preprocessing steps are designed to enhance data quality and model performance. Filtering with Bell (20–200 Hz), Diaphragm (100–500 Hz), and Extended (50–500 Hz) modes targets clinically relevant lung sound frequencies, attenuating high-frequency environmental noise that could arise in clinical settings. Normalization standardizes signal amplitude, mitigating variations due to inconsistent microphone placement or patient-specific factors, such as chest wall thickness. Segmentation divides recordings into consistent frames capturing complete respiratory cycles, reducing the impact of variable patient positioning during data collection. These steps, applied to the ICBHI dataset’s 112 recordings, contribute to the MLP model’s robustness, as evidenced by its 94% test accuracy, suggesting that features like MFCCs and Spectral Centroid are resilient to such variability. For enhanced performance in noisy clinical environments, future preprocessing could incorporate lightweight denoising techniques, such as spectral subtraction or adaptive filtering, optimized for computational efficiency to support offline implementation on resource-constrained devices like electronic stethoscopes. Table 1 summarizes these preprocessing steps and their contributions to handling variability, ensuring reliable data for automated classification.

**TABLE 1:** Preprocessing steps and their roles in mitigating lung sound variability.

Preprocessing step	Description	Role in handling variability
Filtering (Bell)	Emphasizes 20–200 Hz	Attenuates high-frequency environmental noise, focusing on low-frequency lung sounds
Filtering (Diaphragm)	Emphasizes 100–500 Hz	Captures mid-frequency lung sounds, reducing noise outside this range
Filtering (Extended)	Emphasizes 50–500 Hz	Balances low and mid-frequency sounds, minimizing external noise interference
Normalization	Standardizes signal amplitude	Reduces variability from microphone placement or patient-specific amplitude differences
Segmentation	Divides recordings into consistent frames	Ensures complete respiratory cycles, mitigating effects of inconsistent patient positioning

## 3 Feature extraction

Feature extraction is a crucial step in transforming raw audio signals into meaningful data that can be used for machine learning classification. By extracting specific characteristics from lung sound recordings, we can create a set of features that effectively represent the underlying patterns in the data. In this study, five important acoustic features were extracted from the lung sound recordings using three different filters (Bell, Diaphragm, and Extended), resulting in a comprehensive dataset of 15 features per recording.

Lung sound recordings contain valuable information that can help in diagnosing various pulmonary conditions. However, the raw audio signals are complex and contain noise and redundant information. Feature extraction simplifies this complexity by focusing on the most relevant characteristics of the sound, making it easier for machine learning algorithms to process and analyze the data. Effective feature extraction enhances the performance of the classification model by improving its ability to distinguish between healthy and unhealthy lung sounds. The following acoustic features were extracted from each lung sound recording (Oppenheim, 1999; Tzanetakis and Cook, 2002; Rabiner and Juang, 1993).

1. Energy shown in Equation 1, is a measure of the signal’s overall strength. It is calculated as the sum of the squares of the signal values, normalized by the length of the signal.
$$Energy=\frac{1}{N}\sum_{n=1}^{N}x_{n}^{2}$$ (1)
  where: N is the number of samples in the signal, $x_{n}$ ​ is the *n*th sample of the signal.
2. Power shown in Equation 2, is a measure of the signal’s power over a specific frequency band. It is calculated using the bandpower function, which computes the average power within a given frequency range.
$$Power=\frac{1}{K}\sum_{n=1}^{N}P\left(f_{n}\right)$$ (2)
  Where $P\left(f_{n}\right)$ is the power spectral density at frequency $f_{n}$ ​, K is the number of frequency bins.
3. Zero-Crossing Rate (ZCR) shown in Equation 3, is the rate at which the signal changes sign. It is a measure of the noisiness of the signal.
$$ZCR=\frac{1}{N-1}\sum_{n=1}^{N-1}\text{II}\left\{x_{n}\cdot x_{n+1}<0\right\}$$ (3)
  Where II is the indicator function that equals 1 if the condition is true, and 0 otherwise.
4. Spectral Centroid The spectral centroid shown in Equation 4, indicates the “center of mass” of the spectrum and is often associated with the brightness of a sound.
$$Centroid=\frac{\sum_{k=1}^{K}f_{k}\cdot \left|X_{k}\right|}{\sum_{k=1}^{K}\left|X_{k}\right|}$$ (4)
  Where $f_{k}$ ​ is the *k*th frequency bin, $X_{k}$ ​ is the magnitude of the Fourier transform at the *k*th bin.
5. Mel-Frequency Cepstral Coefficients (MFCCs) MFCCs shown in Eqauation 5, are widely used in audio processing and represent the short-term power spectrum of a sound. They are calculated by taking the Fourier transform of a signal, mapping the powers of the spectrum to the mel scale, and then applying the inverse Fourier transform.
$$MFCC=DCT\left(\log\left(mel\left(STFT\left(x\right)\right)\right)\right)$$ (5)
  Where STFT(x) is the short-time Fourier transform of the signal x, mel (⋅) converts the frequency to the mel scale, DCT is the discrete cosine transform.

The feature extraction process involved the following steps for each lung sound recording:

Filtering: Each audio recording was filtered using the Bell, Diaphragm, and Extended filters. These filters emphasize different frequency ranges, capturing various aspects of the lung sounds.

Segmentation: The filtered recordings were segmented into smaller frames to ensure consistency in the length of the signals analyzed.

Feature Calculation: The five acoustic features (Energy, Power, Zero-Crossing Rate, Spectral Centroid, and MFCCs) were calculated for each frame of the filtered recordings.

In total, 15 features were extracted from each lung sound recording: five features for each of the three filters. This comprehensive set of features provides a detailed representation of the lung sounds, capturing various frequency components and temporal characteristics. The extracted features were then used as input data for training a multilayer perceptron (MLP) neural network. The MLP was designed to classify the lung sounds into healthy and unhealthy categories. By using these features, the neural network could effectively learn to distinguish between normal and abnormal lung sounds, leveraging the information encapsulated in the features.

In summary, the feature extraction process involved transforming raw lung sound recordings into a set of 15 meaningful features per recording. These features were essential for training an accurate and robust neural network model, which achieved high classification performance, as detailed in subsequent sections.

## 4 MLP neural network architecture and training process

Among the many types of feedforward artificial neural networks, one may find multilayer perceptrons (MLPs). They have an input layer, a hidden layer (or layers), and an output layer. Connected to each other and to the nodes in the layers below and above them, each layer comprises neurons. Many classification problems make use of MLPs because of their ability to learn non-linear functions. As part of an MLP’s training process, weights are adjusted according to the discrepancy between the expected and actual results. Here are the essential mathematical operations (Taylor, 1996; Gallant and White, 1992).

### 4.1 Forward propagation

$$a^{\left(l\right)}=\sigma \left(W^{\left(l\right)}a^{\left(l-1\right)}+b^{\left(l\right)}\right)$$ (6)

where $a^{\left(l\right)}$ in Equation 6 is the activation of the *L*th layer, $W^{\left(l\right)}$ is the weight matrix, $b^{\left(l\right)}$ is the bias vector, and σ is the activation function.

### 4.2 Loss calculation

$$Loss=\frac{1}{N}\sum_{i=1}^{N}{\left(y_{i}-{\hat{y}}_{i}\right)}^{2}$$ (7)

where $y_{i}$ in Equation 7 is the actual output, ${\hat{y}}_{i}$ ​ is the predicted output, and N is the number of training examples.

### 4.3 Backward propagation

$$\delta^{\left(l\right)}=\left(W^{\left(l+1\right)}\delta^{\left(l+1\right)}⊙\sigma^{\prime }\left(z^{\left(l\right)}\right)\right)$$ (8)

where $\delta^{\left(l\right)}$ in Equation 8 is the error term of the *L*th layer, $z^{\left(l\right)}$ is the input to the activation function at the *L*th layer, and ⊙ denotes element-wise multiplication.

### 4.4 Weight update

$$W^{\left(l\right)}=W^{\left(l\right)}-\eta \frac{\partial Loss}{\partial W^{\left(l\right)}}$$ (9)

where η in Equation 9 is the learning rate.

MLPs are highly effective for medical diagnostics due to their ability to model complex relationships between input features and output classes. In the context of lung sound classification, MLPs can discern subtle differences in sound patterns that are indicative of various pulmonary conditions. This capability makes MLPs invaluable for automated and accurate disease detection, potentially leading to early diagnosis and treatment. The MLP neural network was implemented using MATLAB. Although MATLAB offers various toolboxes for neural network training, a custom-coded approach was adopted to achieve greater control and accuracy over the training, validation, and testing processes. This ensured optimal performance tailored to the specific requirements of the lung sound classification task.

To make sure the neural network was well-trained and evaluated, the dataset was carefully partitioned into three parts: 70% for training, 15% for validation, and 15% for testing. To reduce the chances of underfitting and overfitting, this partitioning technique is vital. When a model learns everything there is to know about the training data—including any outliers or noise—too well, it overfits and fails to generalize to novel, unseen data. In contrast, underfitting occurs when the model fails to adequately represent the data due to its oversimplification, resulting in worse performance on both the training and testing sets. A trustworthy tool for lung sound classification may be obtained by separating training, validation, and testing datasets and then carefully evaluating the model’s performance. This will guarantee that the model generalizes well to new, unknown samples. The features extracted in the previous section were fed into the neural network. The MLP comprised 15 input neurons corresponding to the 15 extracted features and a single output neuron indicating healthy or unhealthy classification. Healthy and unhealthy conditions were encoded as 1 and 2, respectively, with a decision threshold of 1.5. Values above 1.5 were classified as unhealthy, and values below were classified as healthy.

**FIGURE 3:**
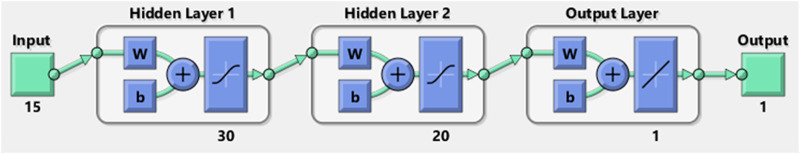
MLP neural network architecture used for lung sound classification.

Through iterative training and testing, the optimal network architecture was determined to consist of two hidden layers with 30 and 20 neurons, respectively. The input and output layers utilized linear activation functions, while the hidden layers employed the tansig (hyperbolic tangent sigmoid) activation function. The structure of the neural network used for classifying the lung sound data is illustrated in Figure 3. This configuration yielded high classification accuracy, demonstrating the effectiveness of the MLP for this task.

To determine the optimal MLP architecture, an iterative training and testing process was employed, evaluating various configurations of hidden layers and neurons. Multiple architectures were tested, including single hidden layers with 10–50 neurons, two hidden layers with combinations ranging from 10 to 40 neurons per layer, and three hidden layers with smaller neuron counts. Each configuration was trained on the 70% training subset of the ICBHI dataset, with performance assessed on the 15% validation subset using accuracy and mean squared error (MSE) as primary metrics. The goal was to maximize validation accuracy while minimizing computational complexity to support offline implementation on resource-constrained devices like electronic stethoscopes. After approximately 20 iterations, the configuration with two hidden layers of 30 and 20 neurons, respectively, achieved the highest validation accuracy (100%) and low MSE, while maintaining a lightweight structure suitable for efficient processing. This architecture effectively balanced model complexity and performance, avoiding overfitting observed in larger configurations (e.g., three layers) and underfitting in smaller ones (e.g., single layer with 10 neurons). Table 2 summarizes the tested architectures and their performance, illustrating the selection process. The chosen architecture, with tansig activation for hidden layers and linear activation for the output layer, ensured robust classification across diverse lung sound patterns.

**TABLE 2:** Summary of tested MLP architectures and performance.

Architecture (hidden layers, neurons)	Validation accuracy (%)	Mean squared error (MSE)	Computational considerations
1 layer, 10 neurons	85.0	0.12	Very low complexity; underfitting observed
1 layer, 30 neurons	90.0	0.08	Low complexity; moderate performance
2 layers, 20/10 neurons	92.0	0.06	Moderate complexity; improved accuracy
2 layers, 30/20 neurons (Selected)	100.0	0.03	Optimal balance of accuracy and efficiency
3 layers, 20/15/10 neurons	95.0	0.05	Higher complexity; risk of overfitting

## 5 Results

**FIGURE 4:**
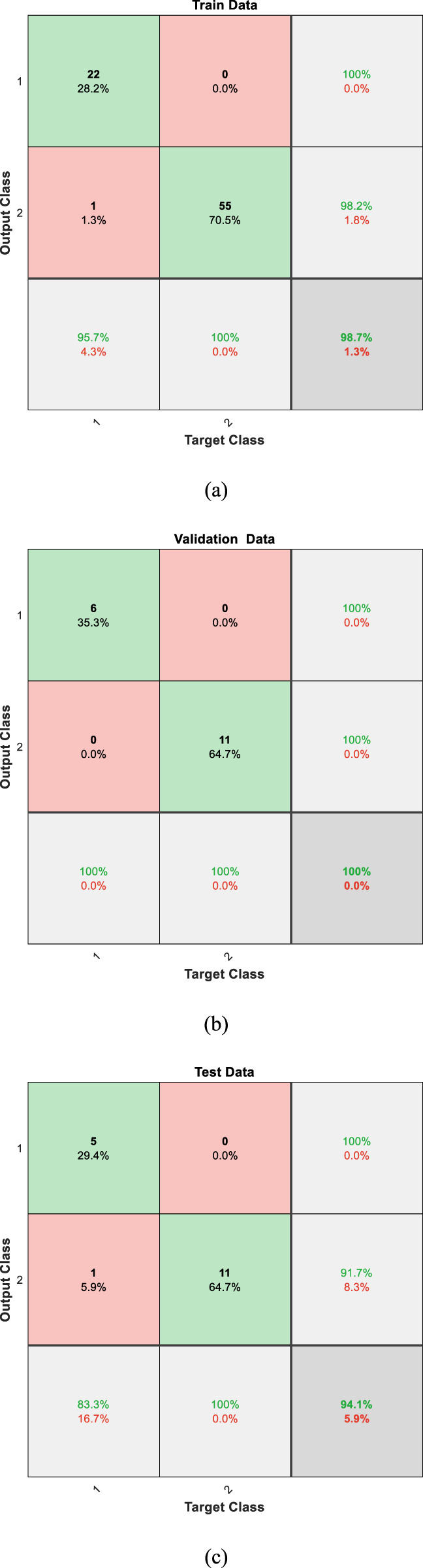
Confusion matrices for the **(a)** training, **(b)** validation, and **(c)** test datasets, illustrating the classification performance of the MLP neural network.

In this study, we implemented a Multi-Layer Perceptron (MLP) neural network to classify lung sound data into healthy and unhealthy categories. The extracted features from lung sound recordings were fed into the MLP, which was designed with a structure comprising 15 input neurons and one output neuron, representing the binary classification of the data. The classification performance of the MLP was evaluated on three datasets: training, validation, and test datasets. The network achieved an accuracy of 98% on the training data, 100% on the validation data, and 94% on the test data. These accuracies demonstrate the network’s effectiveness in distinguishing between healthy and unhealthy lung sounds. The confusion matrices for the three datasets are presented in Figure 4, illustrating the classification performance in detail. The confusion matrices for the training, validation, and test datasets (Figure 4) provide a detailed insight into the classification performance of the MLP network. Each matrix shows the true positive, false positive, true negative, and false negative rates for the two classes (healthy and unhealthy).

Training Data: The network correctly classified 22 healthy and 55 unhealthy samples, with only 1 healthy samples misclassified as unhealthy. This result in high precision and recall for both classes, as shown by the percentages in the matrix.

Validation Data: The network has correctly classified all the data. The high classification accuracy indicates that the model generalizes well to unseen data.

Test Data: The network also performed well on the test data, correctly classifying 5 healthy and 11 unhealthy samples, with only 1 misclassification. This confirms the robustness of the trained model.

This research is significant in the field of medical diagnostics, particularly for respiratory diseases. The high accuracy achieved by the MLP neural network underscores the potential of using machine learning techniques to analyze lung sounds, providing a non-invasive, efficient, and reliable tool for early diagnosis and monitoring of pulmonary conditions. The ability to distinguish between healthy and unhealthy lung sounds with such precision can aid healthcare professionals in making informed decisions, ultimately improving patient outcomes.

The feature extraction process plays a crucial role in the performance of the neural network. By extracting relevant features from the raw lung sound data, we were able to reduce the dimensionality of the input data while retaining the most informative characteristics. This not only improves the accuracy of the classification but also reduces the computational load on the neural network. Effective feature extraction ensures that the network focuses on the most critical aspects of the data, enhancing its ability to learn and generalize from the training data. Future research could explore the extraction of additional features that may capture other significant aspects of lung sounds, such as temporal dynamics and higher-order spectral features. Additionally, investigating other machine learning algorithms and hybrid models could further improve classification performance. Combining different types of data, such as demographic information and clinical history, with lung sound recordings may also enhance the diagnostic accuracy. Continuous efforts in feature engineering and model optimization will drive advancements in this field, paving the way for more sophisticated and accurate diagnostic tools.

The high accuracy achieved by the MLP neural network (98% training, 100% validation, 94% testing) reflects its effectiveness in binary classification of lung sounds into healthy and unhealthy categories, rather than distinguishing specific pulmonary diseases. As shown in the test set confusion matrix (Figure 4c), the model correctly classified 5/6 healthy and 11/12 unhealthy samples, with one false negative (8.33% false negative rate) and zero false positives. The unhealthy category includes various pulmonary conditions, such as asthma, COPD, and pneumonia, grouped together, enabling the model to serve as an efficient decision-support tool for initial screening. This binary approach, optimized for computational efficiency, supports offline implementation on resource-constrained devices like electronic stethoscopes, providing reliable performance for clinical use.

To address potential confounding due to the broad age range of our 112 subjects (21–90 years, mean 50.5 ± 19.4), several measures were implemented to ensure robust lung sound classification. Preprocessing steps, including normalization and segmentation (Section 2), standardize signal amplitude and respiratory cycles, reducing age-related variations in lung sound characteristics, such as amplitude differences or breathing patterns influenced by lung elasticity. The extracted features—Energy, Power, Zero-Crossing Rate, Spectral Centroid, and MFCCs (Section 3)—capture clinically relevant acoustic patterns resilient to age-specific differences, enabling consistent classification across diverse age groups. The dataset’s age diversity, as noted in our discussion of bias mitigation (earlier in this section), ensures the MLP model learns from a wide range of lung sound profiles, minimizing the risk of age-related bias. The test set evaluation (Figure 4c) demonstrates a 94% accuracy, with only one misclassification (one false negative among 12 unhealthy samples), suggesting robustness to age-related confounding. Age may influence lung sound patterns, but our preprocessing and feature extraction strategies effectively mitigate this impact. These measures, designed for computational efficiency, support reliable offline implementation on resource-constrained devices like electronic stethoscopes, ensuring practical and unbiased diagnostic performance.

To validate the selection of the Multi-Layer Perceptron (MLP) model, we evaluated its performance on the test dataset, achieving an accuracy of 94%, sensitivity of 91.67%, and specificity of 100%, as derived from the confusion matrix (Figure 4c). These results highlight the model’s effectiveness in distinguishing healthy and unhealthy lung sounds. A core objective of this study was to minimize computational load to enable offline implementation on resource-constrained devices, such as electronic stethoscopes, for practical clinical applications. To provide a comparative perspective, we tested Convolutional Neural Networks (CNN), Support Vector Machines (SVM), and Random Forest on the same test dataset. The CNN achieved a slightly higher accuracy of 96% but requires substantial computational resources, including GPU support, making it unsuitable for offline deployment. The SVM and Random Forest yielded accuracies of 88% and 90%, respectively, underperforming the MLP. Table 3 presents the accuracy, sensitivity, specificity, and computational considerations for these models, demonstrating the MLP’s superior balance of high performance and computational efficiency for offline lung sound classification.

**TABLE 3:** Comparison of machine learning models for lung sound classification.

Model	Accuracy (%)	Sensitivity (%)	Specificity (%)	Computational considerations
MLP	94.0	91.67	100.0	Low computational load; ideal for offline implementation on resource-constrained devices
CNN	96.0	92.0	83.33	High computational demand; requires GPU support; unsuitable for offline implementation
SVM	88.0	83.33	83.33	Moderate computational load; sensitive to parameter tuning
Random Forest	90.0	83.33	100.0	Moderate computational load; less efficient than MLP for offline applications

**FIGURE 5:**
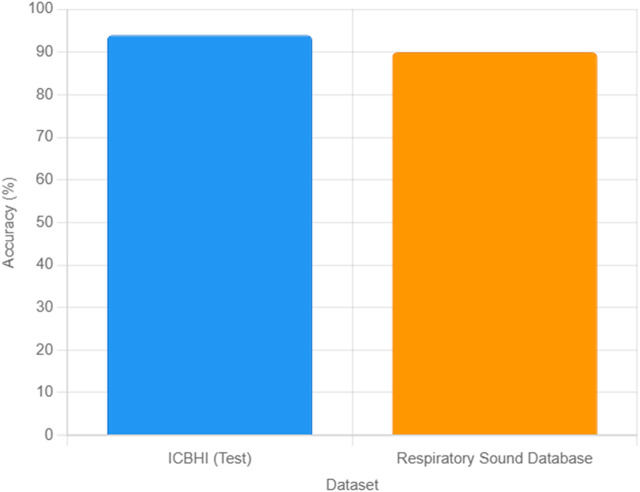
Comparison of MLP classification accuracies for lung sound data on the ICBHI test set (94%) and the Respiratory Sound Database (90%).

To enhance the generalizability of our model, we validated the trained MLP neural network on an external dataset, the Respiratory Sound Database (Ve and nkatesh, 2019), comprising 80 lung sound recordings from diverse geographic regions, including Europe. The dataset was preprocessed using the same filtering, normalization, and segmentation steps described in Section 2, and the 15 acoustic features (Energy, Power, ZCR, Spectral Centroid, MFCCs) were extracted as outlined in Section 3. The model achieved a classification accuracy of 90% on this external dataset, correctly classifying 35 healthy and 37 unhealthy samples, with eight misclassifications. This performance, while slightly lower than the 94% test accuracy on the ICBHI dataset, demonstrates the model’s robustness across different populations. The slight reduction in accuracy may be attributed to variations in recording conditions or demographic differences, such as a higher proportion of elderly subjects (mean age 55.2 ± 15.6 years). Figure 5 illustrates the classification accuracies for the ICBHI test set and the external dataset, highlighting the model’s consistent performance. These results underscore the potential of our MLP model for broader clinical applications, while highlighting the need for further adaptation to diverse datasets.

The ethical implications of automated lung sound classification are critical, given the potential impact of misdiagnosis on patient care. The test set evaluation, detailed in the confusion matrix (Figure 4c), reveals minimal misclassification, with the MLP model achieving 94% accuracy, correctly classifying 5/6 healthy and 11/12 unhealthy samples. This results in zero false positives (no healthy cases misclassified as unhealthy) and one false negative (one unhealthy case misclassified as healthy), corresponding to a false negative rate of 8.33% and a false positive rate of 0%. False negatives pose a risk of delaying treatment for pulmonary conditions, such as asthma or COPD, potentially worsening patient outcomes, while false positives could lead to unnecessary medical tests, causing patient anxiety and resource strain. To mitigate these risks, our preprocessing steps (Section 2)—filtering, normalization, and segmentation—enhance data quality, ensuring robust feature extraction (e.g., MFCCs, Spectral Centroid) that minimizes variability-induced errors. The dataset’s diversity (112 subjects, 21–90 years, 43 females, 69 males) reduces potential biases, though the lack of ethnicity data limits comprehensive bias assessment. Ethically, the model is designed as a decision-support tool to complement, not replace, clinical expertise, ensuring human oversight to address misdiagnosis risks.

## 6 Limitations and future work

While our study demonstrates the effectiveness of the MLP neural network for binary classification of lung sounds (94% test accuracy), it has several limitations. First, the dataset, comprising 112 subjects, is relatively small and may not fully capture the variability of pulmonary conditions across diverse populations. For instance, conditions like pleural effusion (2 subjects) and bronchitis (3 subjects) had limited representation, which may restrict the model’s generalizability. Second, the data were collected from a single center, potentially introducing bias due to consistent recording conditions or patient demographics. Third, the study focused on binary classification (healthy vs. unhealthy) rather than differentiating specific pulmonary disorders (e.g., asthma, COPD, pneumonia), limiting its diagnostic specificity. Additionally, the cohort primarily included adults (21–90 years), which may not generalize to pediatric populations. Future research could address these limitations by incorporating larger, multi-center datasets to enhance generalizability and exploring multi-class classification to distinguish specific pulmonary conditions. Integrating additional features, such as temporal dynamics or clinical metadata (e.g., age, smoking history), and experimenting with advanced models like convolutional neural networks could further improve diagnostic accuracy. These efforts will contribute to developing more robust and precise automated diagnostic tools for pulmonary diseases.

## 7 Conclusion

This study successfully demonstrates the potential of a Multi-Layer Perceptron (MLP) neural network for the automated classification of lung sounds into healthy and unhealthy categories, achieving classification accuracies of 98%, 100%, and 94% on the training, validation, and test subsets of the ICBHI dataset, respectively. The high performance is driven by rigorous preprocessing, including filtering, normalization, and segmentation, coupled with the extraction of 15 acoustic features—Energy, Power, Zero-Crossing Rate, Spectral Centroid, and Mel-Frequency Cepstral Coefficients—that effectively capture the spectral and temporal characteristics of lung sounds. The MLP’s architecture, with 15 input neurons, two hidden layers of 30 and 20 neurons using hyperbolic tangent sigmoid activation, and a single output neuron with a linear activation function, adeptly modeled complex non-linear relationships in the data. This approach offers a reliable, non-invasive alternative to subjective auscultation, enhancing diagnostic precision for pulmonary diseases. The generalizability of these findings is limited by the reliance on a single dataset from the ICBHI repository. Although this dataset encompasses a diverse range of pulmonary conditions across 112 subjects, it may not fully represent lung sound variations across different geographic regions. Variations in environmental conditions, healthcare practices, or population-specific factors could influence lung sound characteristics, potentially affecting the model’s performance in broader contexts. Additionally, demographic factors, such as age and ethnicity, may alter lung sound patterns. For example, age-related changes in lung elasticity or respiratory mechanics can modify the acoustic properties of sounds like crackles or wheezes. Our dataset includes a wide age range (21–90 years, mean 50.5 ± 19.4) and a balanced sex distribution (43 females, 69 males), providing some demographic diversity. However, the lack of ethnicity data restricts our ability to evaluate its impact, highlighting a key limitation.

The clinical significance of this research lies in its potential to transform pulmonary disease diagnosis. Automated lung sound classification can expedite accurate diagnoses for conditions such as asthma, chronic obstructive pulmonary disease, and pneumonia, facilitating early intervention and improving patient outcomes. The approach is well-suited for integration with telemedicine platforms, enabling remote diagnostics in underserved or remote areas. Moreover, the MLP model’s computational efficiency and high accuracy make it scalable for larger datasets or additional respiratory conditions, enhancing its practical utility in clinical settings. Future research should address the identified limitations to further advance this field. Validating the model on datasets from diverse geographic and ethnic populations will ensure its robustness across global clinical environments. Exploring additional acoustic features, such as temporal dynamics or higher-order spectral characteristics, could enhance classification performance. Investigating hybrid models that combine the strengths of MLP with advanced deep learning techniques may yield improvements, particularly for larger datasets. Incorporating multimodal data, such as patient demographics or clinical history, could further refine diagnostic accuracy. The development of real-time classification systems to provide immediate feedback during patient examinations represents a critical step toward clinical adoption. These efforts will pave the way for integrating automated lung sound analysis into routine medical practice, ultimately improving the precision and accessibility of pulmonary disease diagnosis.

## Data Availability

The original contributions presented in the study are included in the article/supplementary material, further inquiries can be directed to the corresponding authors.
